# Maternal and paternal carriage of the annexin A5 M2 haplotype: a possible risk factor for recurrent implantation failure (RIF)

**DOI:** 10.1007/s10815-020-01978-1

**Published:** 2020-11-24

**Authors:** Nina Rogenhofer, Arseni Markoff, Xenia Ennerst, Nadja Bogdanova, Christian Thaler

**Affiliations:** 1 Division of Gynecological Endocrinology and Reproductive Medicine, Department of Gynaecology and Obstetrics, University Hospital of the Ludwig-Maximilians-University, Marchioninistrasse 15, 81377 Munich, Germany; 2 Institute of Human Genetics, UKM and WWU, Muenster, Germany

**Keywords:** M2 Haplotype, Annexin, Recurrent implantation failure

## Abstract

**Objective:**

This study was carried out to determine the potential role of the *M2/ANXA5* haplotype as a risk factor for recurrent implantation failure (RIF). Carriage of the *M2/ANXA5* haplotype that induces prothrombotic changes has been implicated in failure of early pregnancies and placenta-mediated complications (preeclampsia, IUGR, preterm birth).

**Material and methods:**

In the present case control study, 63 couples (females and males) with RIF presenting for IVF/ICSI to the Fertility Center of [masked] were analyzed. RIF was defined as ≥ 4 consecutive failed ART-transfers of ≥ 4 blastocysts or ≥ 8 cleavage-stage embryos of optimal quality and maternal age ≤ 41. Fertile female controls (*n* = 90) were recruited from the same center. Population controls (*n* = 533) were drafted from the PopGen biobank, UKSH Kiel.

**Results:**

Couples carrying the M2/ANXA5 haplotype turned out to have a significantly increased relative risk (RR) for RIF. Compared with female fertile controls, RR was 1.81 with *p* = 0.037 (OR 2.1, 95%CI 1.0–4.3) and RR was 1.70, with *p* = 0.004 (OR 2.0, 95%CI 1.2–3.1) compared with population controls (15.4% M2 carriers). Male partners were comparable with RIF females for M2/ANXA5 haplotypes (28.6% vs. 23.8%, *p* = 0.54). RIF females compared with population controls had a RR of 1.55 (*p* = 0.09) and RIF males compared with population controls had a RR of 1.9 (*p* = 0.01). Couples with ≥ 7 failed transfers showed a RR of 1.82 (*p* = 0.02) compared with population controls.

**Conclusion:**

Our findings suggest that maternal as well as paternal M2/ANXA5 haplotype carriages are risk factors for RIF. These results allow new insights into the pathogenesis of RIF and might help to identify relevant risk groups.

## Introduction

Recurrent implantation failure (RIF) is determined when morphologically good quality embryos repeatedly fail to implant after numerous IVF/ICSI treatment attempts [1–5].

There are several variations to the criteria for defining RIF [2]. Some definitions include the numbers of failed ART cycles and the numbers of transferred embryos. For example, Polanski et al. assess RIF as ≥ 2 consecutive unsuccessful transfers with ≥ 4 embryos or 2 blastocysts of high quality [6]. Others include female age as an additional criterion [1, 3–7], such as the European Society for Human Reproduction and the Embryology (ESHRE) PGD Consortium [4, 5]. They define RIF as ≥ 3 transfers in women under 37 years or, respectively, ≥ 2 transfers in women above 37 years after transfers of 10 good quality embryos. Considering the current success rate of an ART attempt, the number and quality of embryos transferred, and the female age, we defined RIF as ≥ 4 failed consecutive ART transfers of ≥ 4 blastocysts or ≥ 8 cleavage stage embryos of good quality in women ≤ 41 years.

Human implantation is a complex process requiring synchrony between a healthy embryo that should have the potential to implant and a functionally competent and receptive endometrium [8]. A successful development is the result of a coordinated “cross-talk” between the embryo and the endometrium, which leads to the apposition, attachment, and invasion of the embryo into the receptive uterine stroma [2, 8]. Any abnormality will result in an implantation failure.

Despite extensive efforts and research on fundamental causes for RIF, the understanding of this condition is still limited [9]. Frequently, the embryo and its ploidy status have been considered [9, 10]. Failure of implantation due to embryonic causes is associated with either genetic abnormalities or other factors that impair the embryo to develop in utero, to hatch, and to implant [2, 3]. Likewise, maternal factors were suggested to play an essential role for RIF [1–3, 10]. For example, uterine abnormalities [2, 11, 12], thrombophilias [3, 13–16], immunological factors [1–5], the antiphospholipid syndrome, parental genetic disorders [3, 10], and endometrial factors, particularly the receptivity with the window of implantation, were proposed [4, 17–22]. Nevertheless, the majority of RIF cases still remain unexplained [1, 2, 10].

Since 2007, much work has been accumulated on a newly identified hereditary thrombophilic genetic factor, the ‘M2 haplotype’ of the annexin A5 (*ANXA5*) gene. Indeed, we and others identified the M2 haplotype associated with obstetric pathologies such as gestational hypertension, preeclampsia [23–27], fetal growth restriction, preterm birth [26, 28–30], antiphospholipid syndrome [31], and most incriminatory recurrent pregnancy losses (RPL) [32]. Moreover, in our previous studies, we confirmed risks of maternal *M2/ANXA5* haplotype and identified the paternal carrier status as an equal risk factor for RPL and obstetric complications [23, 33].

The *M2/ANXA5* haplotype results in a reduced expression of ANXA5 products usually expressed at the apical surface of the syncytiotrophoblast (SCT) [25, 26, 34], facing the intervillous space and floated with maternal blood [35]. Thus, ANXA5 accomplishes its anticoagulant function necessary for the hemodynamic balance in the placenta and pregnancy, thereby decreasing phospholipid availability for the cascade of coagulation factors [36–39]. As a successful implantation requires a balance of pro- and anticoagulatory mechanism at the embryo-maternal interface and within the placenta, ANXA5 is indispensable for the process of implantation. Anti-annexin A5 antibodies (anti-ANXA5) have been identified to influence and even inhibit the essential functions of the ANXA5, particularly affecting implantation. Matsubayashi et al. demonstrated that RIF patients produce anti-ANXA5 significantly more often than controls (8.3% vs. 1.1%, *p* < 0.05) and consequently postulate anti-ANXA5 antibodies as risk factor for RIF [40]. Currently, there is only one multicenter study by Fishel et al. analyzing the *M2/ANXA5* haplotype in infertility couples undergoing ART procedure [41]. They reported a M2 carrier status of 44% in IVF couples (*n* = 157; one or both partners), 24% of females, 26% of males, and 37% of couples with unexplained infertility [41]. In spite of these intruiging results, the relevance of *M2/ANXA5* for RIF has not yet been studied.

Therefore, the primary objective of this study was to investigate, whether the maternal and/or the paternal *M2/ANXA5* carriage are risk factors for RIF. As a second objective, we aimed to find new insights into the pathogenesis of RIF to identify relevant risk groups. With regard to these goals, we investigated the prevalence of *M2/ANXA5* in a well-defined cohort of RIF couples and compared them to two independent control groups.

## Materials and methods

### Definitions

In this present case control study, RIF was defined as ≥ 4 consecutive failed ART-transfers of ≥ 4 blastocysts or ≥ 8 cleavage-stage embryos of optimal quality and maternal age ≤ 41.

### Study populations and controls

The study population of 63 couples (63 females and 63 males) with a history of recurrent implantation failure was recruited between May 2016 and May 2019 in the Division of Gynecological Endocrinology and Reproductive Medicine of the [masked for blinded review] University. All individuals studied were from European origin, mainly German. After clarification and written informed consent, blood was drawn and DNA was extracted from white blood cells using the QIAmp DNA blood mini kit (Qiagen, Hilden, Germany) and stored in 100-μl aliquots at − 20 °C for further analyses.

**Fig. 1:**
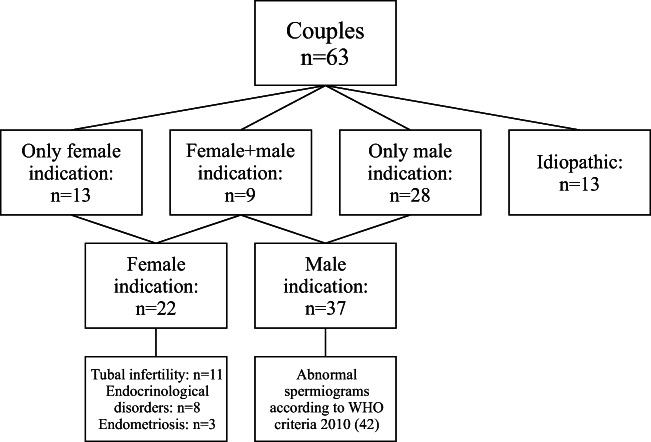
Indications for fertility treatment of the RIF couples subdivided in female and male indications. RIF: recurrent implantation failure. Created with CorelDraw Home&Student X8

Biographic and historic data of RIF couples (RIF females and RIF males) and [masked for blinded review] fertile controls are illustrated in Table 1. The study cohort was composed as follows: 11 couples already terminated the fertility treatment and were contacted for the purpose of this study; 52 couples were still in treatment. Indications for the fertility treatment are illustrated in Fig. 1.

**Table 1:** Biographic and historic data of the RIF couple subclassified in females and males vs. [masked for blinded review] fertile controls

	RIF females (*n* = 63)	RIF males (*n* = 63)	[masked for blinded review] fertile controls	*p*
Age (year)	35 ± 3 (26–41)	39 ± 5 (27–50)	34 ± 5 (21–41)	0.049
Body mass index (kg/m^2^)	23 ± 2 (19 - 30)	-	-	-
Pregnancies (*n*)	0.2 ± 0.5 (0–2)	-	2 (1–5)	< 0.0001
Deliveries (*n*)	0.0 ± 0.2 (0–1)	-	2 (1–5)	< 0.0001
Miscarriages (*n*)	0.1 ± 0.4 (0–2)	---	0	< 0.0001
Ectopic pregnancies (*n*)	0.0 ± 0.2 (0–1)	---	0	< 0.0001

*n* number, *RIF* recurrent implantation failure

In total, 309 embryo transfers (ET) were performed, and altogether, 556 embryos were transferred. Out of the 309 ET, 154 transfers were fresh ET with 155 (53.5%) day 5 transferred blastocysts and 136 (46.7%) day 3 transferred cleavage stages. The remaining 155 transfers were frozen-thawed ET with totally 195 (73.6%) transferred blastocysts (d5 transfers) and 70 (26.4%) cleavage stages (d3 transfers). Regarding the number of transferred embryos, 62 (11.2%) single ET and 494 (88.8%) double ET were carried out.

The study group was compared with two independent control groups:

1. 90 fertile women with inconspicuous timespan to conception (< 10 months), uncomplicated pregnancies, no gestational pathologies, at least one spontaneous term delivery of a healthy normal weight singleton, and no miscarriages. This fertile control group was aged between 18 and 41 years and showed a BMI of 19 to 30 kg/m^2^ according to the study population. They were recruited between May 2016 and May 2019 in the outpatient clinic of the Department of Gynecology and Obstetrics, [masked for blinded review] University ([masked for blinded review] *controls*)*.* Biographic data and gestational history of the [masked for blinded review] controls are shown in Table 1.
2. Previously recruited German control group representing a population control sample drafted from the PopGen biobank at the University Clinic Schleswig-Holstein Kiel (*n* = 533) [42]. The PopGen population controls were from Northwest Germany, federal state of Schleswig-Holstein, and consisted of healthy subjects identified through official population registers. The sample used in this study consisted of about equal numbers of men and women, distributed among the three age groups (18–30, 30–50, 50–80 years).

### Study criteria and RIF screening

The study criteria (inclusion and exclusion criteria) are summarized in Table 2.

**Table 2:** Study criteria

Signed informed consent	
Age ≥ 18 ≤ 41 years	
RIF definition fulfilled	
Unexplained RIF	
European origin	
BMI > 19 < 30 kg/m^2^	
Non-smoker	
No intake of medications for systematic diseases	e.g., anti-epileptics, anti depressives
No infectious diseases	Hepatitis, HIV, tuberculosis

***RIF*** **recurrent implantation failure,** ***BMI*** **body mass index (calculated as weight in kilograms divided by the square of height in meters)**

All patients had undergone an extensive diagnostic work up to identify any potential cause for RIF before study inclusion. If any potential reason was detected, it was eliminated, if this was possible and a benefit could be expected; otherwise patients were excluded. In brief, the following factors were screened: uterine anomalies were clarified by hysteroscopy. Endometrial receptivity analysis (ERA) was carried out by endometrial biopsy in a non-treatment cycle before embryo transfer to identify the window of implantation. Endocrinologic dysfunctions (polycystic ovary syndrome according to the Rotterdam criteria [43], hyperprolactinemia, hyperandrogenemia, thyroidal dysfunctions such as hypo-/hyperthyreosis and thyroid autoantibodies), autoimmune disorders (antinuclear antibodies > 1:240), inherited thrombophilias (factor V-Leiden mutation, the prothrombin 20210G>A), and deficiencies in coagulation factors (protein C, protein S, factor XII, antithrombin) were excluded. An antiphospholipid syndrome was ruled out according to the international consensus statement on an update of the classification criteria for definite antiphospholipid syndrome [44]. Parental genetic disorders were clarified by genetic counseling and karyotype testing of the couples. In case of a chromosomal anomaly, a preimplantation genetic screening (PGS) was offered and carried out after approval by a nominated and qualified ethic committee.

### Ethical approval

The study complied with the ethical guidelines of the institutions involved and was approved of the Review Board of the [masked for blinded review] University (IRB 238-16). Informed consent was obtained from all patients and controls. The criteria of strengthening the reporting of observational studies in epidemiology [45] were observed as far as applicable.

### Genotyping and statistical analysis

The extracted DNA was genotyped through amplicon sequencing as previously described [46]. The genotypes were recorded in table format and coded for further usage. Odds ratios (OR) with 95% confidence intervals (CI) were calculated by using the FREQ procedure intrinsic to the SAS statistical software package, version 9 (SAS Institute, Cary, NC, USA). Departures from the Hardy-Weinberg equilibrium were computed by a Markov Chain Monte Carlo (MCMC) implementation of an exact test, as part of the Genepop package [47].

## Results

In total, 63 couples with RIF were investigated and compared with 90 fertile female controls ([masked for blinded review] controls) and with 533 population controls (PopGen controls). The study group was in Hardy-Weinberg equilibrium for the ANXA5 haplotypes (RIF couples: MCMC *P* = .2090, RIF females: MCMC *P* = .3378, RIF males: MCMC *P* = .4703) as well as both control groups [masked for blinded review] controls (MCMC *P* = .1150) and PopGen controls (MCMC *P* = .409), respectively. The genetic frequency of M2 (Table 3) was found to be considerably higher in RIF patients (RIF couples: 0.143, RIF females: 0.127, RIF males: 0.159) than in [masked for blinded review] controls (0.083) and population controls (0.082).

**Table 3:** Genotype frequencies of ANXA5 gene promoter haplotypes in European RIF couples, RIF females and RIF males, and different control groups

Index	RIF couples		RIF females		RIF males		[masked for blinded review] fertile controls		PopGen controls	
Genotype	Observed	Expected^a^	Observed	Expected^a^	Observed	Expected^a^	Observed	Expected^a^	Observed	Expected^a^
N/N	75 (59.5)	73.8	41 (65.1)	41.2	34 (54.0)	32.8	62 (68.9)	60	415 (77.9)	413.3
N/M1	15 (11.9)	17.7	6 (9.5)	6.5	9 (14.2)	10.9	15 (16.7)	14.8	35 (65.7)	47.8
M1/M1	3 (2.4)	1.0	1 (1.6)	0.2	2 (3.2)	0.8	0	0.8	1 (0.2)	1.5
N/M2, M1/M2^b^	30 (23.8)	31.0	14 (22.2)	14.1	16 (25.2)	17.0	11 ( 12.2)	13.8	77 (14.4)	79.9
M2/M2	3 (2.4)	2.5	1 (1.6)	1.0	2 (3.2)	1.5	2 (2.2)	0.6	5 (0.9)	1.4
Total	126	126	63	63	63	63	90	90	533	533

Note: Values are number (percentage)

*N* wild type, *M1* M1-haplotype, *M2* M2-haplotype

^a^Expected: genotype frequency expected at Hardy-Weinberg equilibrium, calculated with the Genepop package

^b^Genotype M1/M2 was only observed in two RIF males, in three [masked for blinded review] fertile controls and five PopGen controls

M2 carriers presented a 1.8-fold elevated risk for RIF (OR 2.1, 95%, CI 1.0–4.3) compared with noncarriers. In comparison with the PopGen control group (*n* = 533), similar results were obtained (Table 4). Results regarding RR and OR of the M2/ANXA5 carriership of RIF couples, subclassified in RIF females and RIF males compared with [masked for blinded review] fertile controls and PopGen controls, are illustrated in Table 4.

**Table 4:** Relative risks and odds ratios of the M2 carriage of RIF couples, RIF females and RIF males compared with [masked for blinded review] fertile controls and PopGen controls

		RR [95% CI]	OR [95% CI]	*p*
RIF couples	[masked for blinded review] fertile controls	1.81 [1.01–3.25]	2.10 [1.03–4.27]	0.037
	PopGen controls	1.70 [1.19–2.42]	1.95 [1.23–3.10]	0.004
RIF females	[masked for blinded review] fertile controls	1.65 [0.84–3.22]	1.85 [0.81–4.23]	0.141
	PopGen controls	1.55 [0.95–2.51]	1.72 [0.92–3.21]	0.087
RIF males	[masked for blinded review] fertile controls	1.98 [1.05–3.73]	2.37 [1.06–5.29]	0.027
	PopGen controls	1.86 [1.20–2.88]	2.20 [1.21–3.99]	0.008
RIF couples with idiopathic infertility	[masked for blinded review] fertile controls	2.13 [0.99–4.58]	2.63 [0.95–7.29]	0.057
	PopGen controls	2.00 [1.09–3.68]	2.44 [1.03–5.81]	0.043
Higher-grade RIF (≥ 7 failed transfers))	[masked for blinded review] fertile controls	1.94 [0.99–3.79]	2.30 [0.98–5.40]	0.052
	PopGen controls	1.82 [1.12–2.96]	2.14 [1.10–4.14]	0.021

*RIF* recurrent implantation failure, *RR* relative risk, *OR* odds ratio, *CI* confidence interval

RIF couples as well as RIF males showed significantly higher RR and OR for M2/ANXA5 carriership compared with both control groups (RIF couples vs. [masked for blinded review] fertile controls: RR 1.81; *p* = 0.037, respectively, vs. PopGen controls: RR 1.70; *p* = 0.004; RIF males vs. PopGen controls: RR 1.86; *p* = 0.008) (Table 4).

Subanalysis revealed 15 RIF females (1 homozygote) and 18 RIF male partners (2 homozygotes) being M2 carriers (Table 3). Thus, the genetic risk transmitted by maternal and paternal alleles turns out to be comparable. As men were showing higher AF, the sample size was too small (*n* = 63) to draw further conclusions.

Interestingly, RIF couples with idiopathic infertility and RIF couples with higher grade RIF (≥ 7 failed transfers) showed significantly increased RR and OR compared with PopGen controls (RR 2.0, OR 2.44; *p* = 0.043, respectively, RR 1.82, OR 2.14; *p* = 0.021).

Couples with idiopathic infertility presented M2/ANXA5 non-significantly more often than couples with identified indication for ART (30.8% vs. 25.0%, *p* = 0.55).

## Discussion

Our study strongly supports an association of the M2/ANXA5 haplotype with RIF. The obtained results suggest an active role of the haplotype in RIF events. Our findings of equal maternal (23.8%) and paternal (28.6%) M2 carriership suggest embryonic and possibly maternal factors.

Highly ordered 2D arrays of ANXA5 have been found to be indispensable for membrane repair in murine perivascular cells [48, 49]. Further investigations confirmed this finding in human cyto- and syncytiotrophoblasts [50]. Additional studies showed that 2D arrays of ANXA5 are essentially involved in the regulation of trophoblast aggregation to form syncytia. Hence, this 2D network of ANXA5 is essential for successful fusion of cells [51]. Villous cytotrophoblasts fuse throughout pregnancy to form multinucleated syncytia on chorionic villi that extends into the maternal placental blood circulation to form an interphase allowing effective exchange of gases and nutrients in the intervillous chamber [52]. Moreover, these multinucleated syncytia produce and secrete pregnancy-specific hormones [53], such as hCG [54], that are of pivotal importance for implantation, placentation, and subsequently, a successful pregnancy. Also, the cellular fusion and membrane repair events are of importance for a successful embryonal implantation at the forming of the cell-to-cell interphase composed of extraembryonic and endometrial receptive membranes to trigger the necessary programs of early placentation. It would be then logical to propose that paternal (embryonic) and maternal factors would have about equal bearing in these processes. A reduced expression of ANXA5 in carriers of the M2 haplotype may disturb cell fusion and membrane repair ultimately leading to implantation failure.

To our knowledge, this is the first study on the role of *M2/ANXA5* in RIF. Previously, it has been demonstrated that the haplotype was a risk factor for infertility in IVF cohorts [41, 55]. In this context, the authors demonstrated in 2014 that IVF-treated couples (*n* = 157) with infertility had significantly increased M2 carriage, whereas maternal and paternal carriages impose an equal risk [41]. In the light of its importance for patients with RPL and potentially implantation failure, the study has assessed the incidence of carrier status for the *M2/ANXA5* haplotype in both the female and male of couples with at least one failed IVF cycle. In 314 patients (157 couples), 44% of couples (one or both partners), 24% of females, 26% of males, and 37% of couples with unexplained infertility were M2 carriers [41]. This rather high incidence inspired further more dedicated studies on specific patient populations and the value of post embryo-transfer therapy. Couples (*n* = 77) with one or both partners carrying *M2/ANXA5* showed a correlation of this haplotype with an adverse IVF outcome [55]. A pragmatic, multicenter, prospective cohort study of *M2/ANXA5* haplotype screening, and low molecular weight heparin (LMWH) treatment following embryo transfer in 103 IVF couples positive for M2 was conducted. They were compared with a group of 1000 contemporaneous randomly selected unscreened and untreated couples undergoing assisted conception, from which 103 matched control couples were extracted. The primary outcome measure was live birth incidence. Secondary outcomes were results following ET and live birth outcome by gender and M2 carriage and allelic dose influence. In the result, the tested and treated cohort of *M2/ANXA5* carriers reached a similar live birth rate (37.9%) per ET cycle compared with both, the more fertile comparison group (38.5%) and the 103 matched controls (33.0%). Significantly more treated male carrier only couples had a live birth of 58.3% vs. 25.0% (*p* = 0.045) versus female M2. Thus, pragmatic *M2/ANXA5* screening and treatment with LMWH in couples undergoing IVF were associated with similar outcome to couples with more beneficial prognostic factors. The difference in live birth outcome for treated male-only carrier couples may be consistent with an additional maternal thrombophilic factor that may adversely affect pregnancy, although other mechanisms, such as LMWH dosage effect, are feasible. This study suggested that LMWH treatment should be started prior to clinical pregnancy.

A very recent report identified alternative means of supplementing anticoagulation, through elevated ANXA5 expression [56]. Physiological micromolar Zn^2+^ stimulated ANXA5 transcription, raising ANXA5 protein expression and surface abundance on choriocarcinoma human placental cells (BeWo) and human umbilical vein endothelial cells (HUVEC), thus resulting in extended coagulation times. In this study, Zn^2+^-fed AnxA5 functionally deficient pregnant mice exhibited a trend to increase litter size when primiparous that grew similarly to wild-type progeny in subsequent pregnancies. A raised AnxA5 signal upon Zn^2+^ treatment was confirmed in murine placentae. Micromolar Zn^2+^ stimulated ANXA5 expression in cell culture directly and attenuated RPL in AnxA5 genetically deficient mice, without notable toxicity effects.

However, currently, there is no consistent definition of RIF. Some authors include the number of failed ART-cycles and the number of transferred embryos as criteria for defining RIF. Others, additionally, include the female age [1, 3–7, 10]. The ESHRE guideline addresses female age (< 37 years and > 37 years), however, without an upper limit. As clinical pregnancy rate per embryo transfer decreases from 20.8% in women at the age of 41 years to 17.7% at the age of 42 and the rate of aneuploid oocytes increases from about 57% to 68% [57, 58], we included the female age and set the cutoff at the female age of ≤ 41 years. Considering the current success rate of an ART attempt, the number and quality of embryos transferred as well as the female age, we defined RIF as ≥ 4 failed consecutive ART transfers of ≥ 4 blastocysts or ≥ 8 cleavage stage embryos of good quality in women ≤ 41 years. With these more refined RIF criteria, we think we can substantially increase the significance of our study.

Furthermore, to discuss the advantages of our novel approach, we refer to the well-defined study group including 63 infertile couples of 63 women as well as 63 men compared with two control groups: one unselected, large population control of 533 females and males in equal parts and one fertile, female control group of 90 participants. All investigated individuals were from European origin; thus, possible interference caused by ethnic differences was excluded. Additionally, to meet the strict study criteria, all patients had undergone an extensive diagnostic work-up for selecting unexplained RIF, including genetic counseling and karyotype testing. A possible weakness of this study should be mentioned at this point, that routine pre-implantation genetic screening was not performed due to legal restrictions and therefore the genetic ploidy status of the transferred embryos remained unknown.

As a matter of fact, multiple failed cycles can leave couples frustrated and desperate for explanations. It is therefore necessary to determine the etiologies of RIF in order to propose new and beneficial solutions for these patients.

Our findings suggest that maternal and paternal M2/ANXA5 haplotype carriages are risk factors for RIF. These results allow new insights into the pathogenesis of RIF and might help to identify relevant risk groups. Further studies are needed to confirm our assumption. As well, therapeutic options have to be identified and established.

## Conclusion

Deficiency of ANXA5 through maternal or paternal carriership of the M2 haplotype is proposed as a risk factor for recurrent implantation failure.

This finding represents a possible explanation for implantation failure after IVF/ICSI as well as for the absence of spontaneous conceptions. Our results provide new insights into the pathogenesis of RIF and could be used to identify patients at risk.
